# Long-term patient-reported outcomes and quality of life after tibiofemoral knee dislocations in patients with open growth plates: a retrospective case series from a Level 1 trauma center

**DOI:** 10.1007/s00068-026-03309-y

**Published:** 2026-09-03

**Authors:** Pierre Hepp, Yasmin Youssef, Ralf Henkelmann

**Affiliations:** Present Address: Department of Orthopedics, Trauma and Plastic Surgery, Leipzig University Hospital, Liebigstraße 20, 04103 Leipzig, Germany

**Keywords:** Pediatric tibiofemoral dislocation, Multiligament knee injury, Ligament reconstruction, Patient-reported outcome measures

## Abstract

**Background:**

Tibiofemoral dislocations in children and adolescents are rare, and treatment guidelines are primarily based on adult data and algorithms. This case series of four pediatric and adolescent patients aims to add to the limited literature and highlights key aspects of diagnosis, treatment, and patient-reported outcomes.

**Methods:**

We conducted a retrospective-prospective single-center case series of pediatric and adolescent patients (< 18 years, open growth plates) treated for tibiofemoral knee dislocations at a Level 1 trauma center between 2017 and 2019. Clinical, radiographic, and intraoperative data were reviewed. In addition, long-term patient-reported outcomes covering both functional recovery and health-related quality of life were assessed using KOOS, IKDC, and EQ-55 D-L.

**Results:**

Four patients (2 male, 2 female), aged between 12 and 16 years, were included in this case series. The mean follow-up was 7.3 years (range 6 to 8 years). All patients sustained Schenck Type III-L dislocations. Early MRI and vascular imaging identified frequent associated injuries such as meniscal and chondral lesions, avulsion fractures, in one case, peroneal nerve injury. A staged surgical approach was used in two of the four cases. No growth disturbances or deformities were observed in the patients based on the available clinical and radiological follow-up. Functional outcomes ranged from moderate to excellent (KOOS: 59–92%, IKDC: 62–100%), while EQ-5D-5L-derived TTO utility values ranged from 0.36 to 1.00, with persistent psychological distress reported by three patients despite good physical recovery.

**Conclusion:**

Pediatric tibiofemoral dislocations can occur after both high- and low-energy trauma and require timely, individualized management. Staged reconstruction tailored to skeletal maturity appears safe and effective, yielding favorable functional outcomes without growth disturbances during long-term follow-up. However, favorable KOOS and IKDC scores did not uniformly correspond to equivalent health-related quality of life, and three of the four patients reported persistent psychological burden related to the injury. Routine psychosocial screening and support should therefore be integrated into the long-term follow-up of pediatric patients after severe knee injuries.

**Clinical trial number:**

Not applicable.

## Introduction

Tibiofemoral dislocations are rare orthopedic injuries that can have devastating consequences if not adequately and timely recognized and appropriately managed [1–5]. The mean age of injury is generally reported at 32 years [2, 5–7]. They account for less than 0.5% of all joint dislocations. In pediatric patients with open growth plates, they are thought to be even more uncommon than in adults [8]. Notably, complex tibiofemoral dislocations classified as Schenck type III and higher are particularly scarce in pediatric patients but can have substantial implications for the long-term joint function and stability [9, 10]. 

Given the scarcity of longitudinal and high evidence level studies that involve pediatric patients, current treatment approaches draw primarily from adult literature and isolated pediatric and adolescent case reports and case series [11–14]. The cited reports range from single-patient case reports to case series including up to 25 patients; however, these studies generally do not provide outcome data stratified by injury subgroups, such as differing Schenck classifications, thereby limiting the granularity and comparability of reported results. This may not adequately account for the distinct anatomical and physiological characteristics of the developing skeleton. Due to the presumed lower biomechanical resistance of the growth plate compared to knee ligaments, traumatic forces may preferentially be transmitted through the physis, resulting in fracture–dislocation patterns rather than purely ligamentous injuries, as is often observed in skeletally mature individuals [8, 12, 15, 16]. A comprehensive understanding of the distinct epidemiology, injury mechanisms, clinical manifestations, and potential complications of these rare but severe injuries in pediatric patients is essential for accurate diagnosis and effective treatment. Such understanding also allows clinicians to better anticipate functional outcomes, long-term quality of life, and the overall burden associated with these injuries.

In this context, we present a case series of four pediatric patients aged between 12 and 16 years, who sustained tibiofemoral dislocations. This case series aims to contribute valuable data on the clinical features, treatment approaches, and patient-reported outcomes as well as health-related quality of life in this rare but clinically significant patient population.

## Methods

A retrospective–prospective, single-center study was conducted that included all patients aged under 18 years with open growth plates treated for knee dislocations Schenck III or higher between 2017 and 2019. Given that all patients in this cohort were aged 12–16 years, the term “adolescent” is used throughout this manuscript to characterize the study population, whereas “pediatric” is retained where referring to the broader literature encompassing younger children with open growth plates. During the study period, no Schenck Type III-M, IV, or V injuries meeting inclusion criteria were identified; the realized cohort therefore comprised exclusively Schenck Type III-L dislocations. The study was carried out in accordance with the ethical standards of the 1964 Declaration of Helsinki and its later amendments [17]. Ethical approval was obtained from the institutional review board (Ethics Committee of the University of Leipzig, reference number 108/21-ek).

Clinical and demographic data were retrieved from electronic medical records, including injury mechanism, associated injuries, treatment strategies, and complications. Body mass index (BMI) was calculated from documented height and weight at the time of injury and classified according to pediatric percentile standards. Skeletal maturity was assessed based on documented open growth plates on standard radiographs at the time of injury; formal bone-age assessment (e.g., Greulich-Pyle atlas) and calculation of residual growth potential were not performed as part of routine clinical care and could therefore not be provided retrospectively.

All patients underwent standardized initial assessment including clinical examination and radiographic imaging. Magnetic resonance imaging (MRI) was performed to assess intra-articular and soft tissue injuries, and vascular status was evaluated using Doppler ultrasound and/or computed tomography angiography when clinically indicated.

Treatment decisions were made on an individual basis, considering patient age, skeletal maturity, injury pattern, and associated injuries. A staged surgical approach was applied in cases with severe soft tissue injury, neurovascular compromise, need for external fixation, or additional procedures (e.g., meniscal root repair) warranting separate operative stages, whereas single-stage management was reserved for patients without these factors, at the discretion of the treating surgeons. Given the open growth plates in all patients, surgical techniques were adapted where anatomically feasible to minimize physeal violation (e.g., epiphyseal or extra-physeal fixation and soft tissue-based repair techniques), consistent with standard practice for skeletally immature patients.

Postoperative rehabilitation was individualized according to injury pattern and surgical procedure. In general, patients remained non-weight-bearing during the first postoperative week, followed by progressive partial weight-bearing and a gradually increasing range of motion, limited to 90° of knee flexion until the sixth postoperative week. From the seventh week onward, rehabilitation was advanced with stepwise increases in weight-bearing and functional loading.

For the prospective follow-up, all patients and their legal guardians provided written informed consent prior to participation. Patients initially underwent a standardized follow-up evaluation including clinical and radiological assessment at defined time points after treatment. At the time of the present study, all patients were re-contacted via telephone, and patient-reported outcome measures (PROMs) were obtained using structured interviews. Follow-up assessments therefore comprised both objective clinical/radiological evaluation and subjective patient-reported outcomes. Growth disturbance and angular deformity were assessed clinically and radiographically at the interim in-person follow-up; the final long-term outcome assessment reported in this study was based on a structured telephone interview and did not include repeat clinical or radiographic examination.

To evaluate long-term outcomes, validated PROMs were collected during follow-up. These included the International Knee Documentation Committee score (IKDC) [18, 19], the Knee injury and Osteoarthritis Outcome Score (KOOS) [20, 21], and the EuroQol five-dimension questionnaire (EQ-5D-5L) [22]. While KOOS and IKDC primarily capture knee-specific function and symptoms, the EQ-5D-5L additionally assesses general health-related quality of life including a mental health dimension. The EQ-5D-5L descriptive system was converted into a single utility index (Time Trade-Off, TTO), ranging from 0 (a health state equivalent to death) to 1 (perfect health), with higher values indicating better health-related quality of life. The TTO-derived utility index was used as the primary summary measure of health-related quality of life, while the EQ-5D VAS was considered a complementary subjective assessment. At the time of outcome assessment, all patients had reached legal adulthood; therefore, no pediatric-specific or age-adapted questionnaires were used.

Given the extreme rarity of the condition and the small sample size (*n* = 4), only descriptive statistics were applied. Outcomes are presented as individual values and ranges, and no inferential statistical analyses were performed.

## Results

A total of four patients (2 male, 2 female), aged 12, 14, 15 and 16 years, were included in this case series. The mean age at injury was 14.3 years and the average BMI was 31.9 kg/m^2^. All patients had a Schenck Type III-L knee dislocation.

**Fig. 1:**
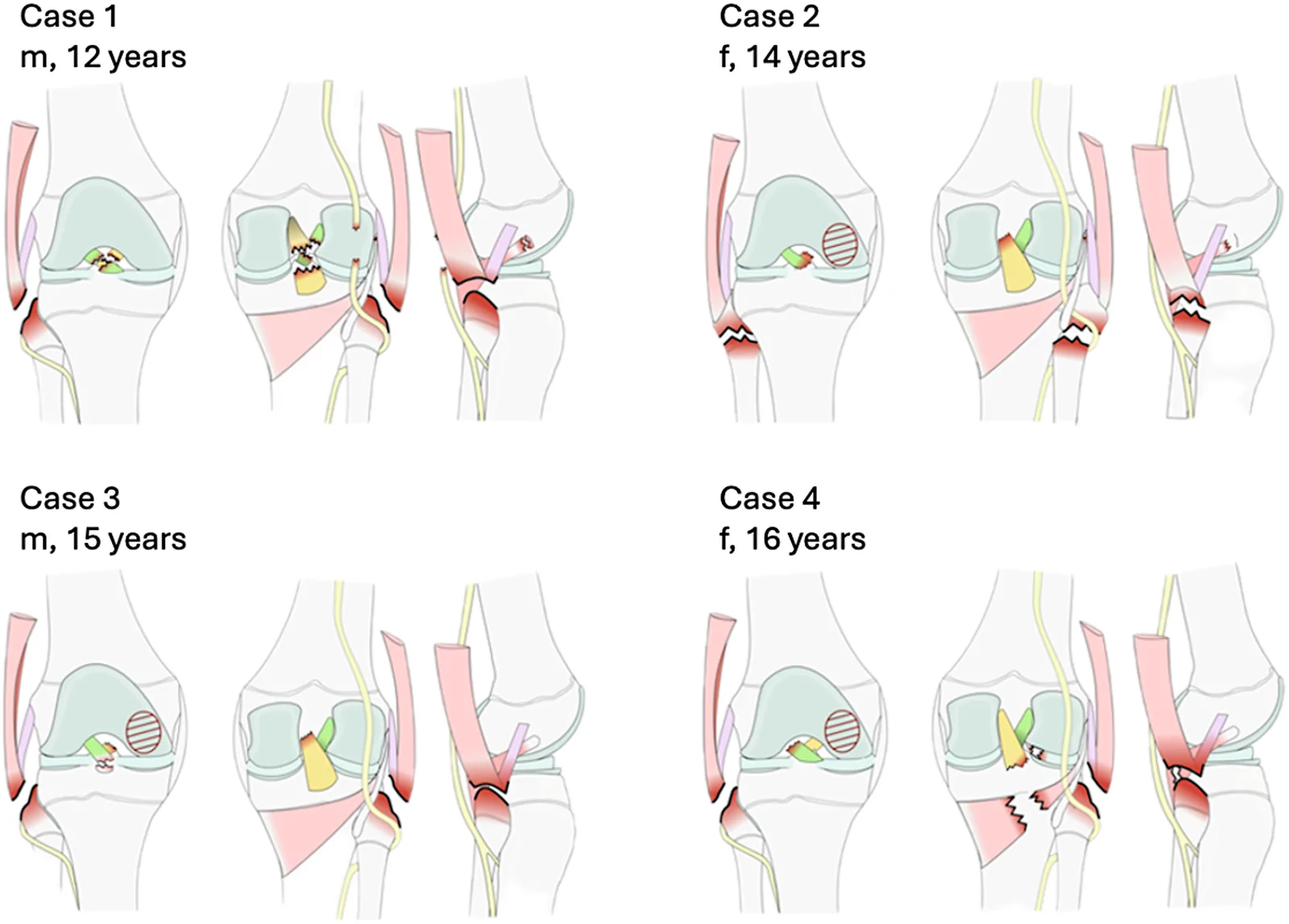
Graphical depiction of the injury mechanisms of the four cases

The dignostic work-up of all patients included conventional x-rays, an MRI, vascular diagnostics (spectral doppler ultrasonography or angiography) and intraoperative stability testing. Individual follow-up times were 8, 8, 7, and 6 years for Cases 1–4, respectively. A summary of the cases including sociodemographic data, exact injury and assessment dates, the injury mechanism, inter-stage surgical intervals, and treatment procedure is shown in Table 1. Figure 1 illustrates the injury mechanisms of the four cases in a graphical format. None of the patients showed a growth disturbance, angular deformities or clinical muscle strength asymmetries at the interim in-person clinical and radiological follow-up. The KOOS score, IKDC and EQ-5D-5L including the Time-Trade-Off (TTO) score at final follow-up are summarized in Table 2.

**Table 1:** Summary of the patients including demographics, injury patterns and performed operative treatment

	Sex	Age at injury	Injury mechanism	Injury	Time to first treatment (days)	Treatment procedure	Time to second-stage procedure (days)
1	male	12	fall during sports class	Schenck Type III lateral: ACL, PCL, LCL complex (fibular), fibular rupture M. biceps, *N*. peroneus damage	2	Single-stage • arthroscopic reconstruction (stitching) of the ACL and PCL, open reconstruction of LCL complex and reattachment of M. bicepsfemoris • secondary microsurgical peroneal nervereconstruction (sural nerve interposition graft)	/
2	female	14	Knee distortion standing on sandy underground	Schenck Type III lateral: ACL, PCL, LCL complex (fibular), avulsion dorsal capsule, impression fracture medial femur condyle	12	Sinle-stage • ligament bracing ACL + PCL and open reconstruction LCL complex	/
3	male	15	Road accident (bike against tractor)	Schenck Type III lateral: ACL, PCL, LCL complex (fibular), impression fracture medial femur condyle	0	Two-stage • external fixation (in external hospital) • ligament bracing PCL, transosseous suture ACL and open reconstruction LCL complex	7
4	female	16	Standing up from crossed-legged sitting	Schenck Type III lateral: ACL, PCL, LCL complex (fibular), avulsion dorsal capsule, fibular rupture M. biceps, impression fracture medial femur condyle, root tear of the medial meniscus	5	Two-stage: • meniscus refixation, open reconstruction of LCL complex and refixation biceps • ACL and PCL reconstruction in second step	221

**Table 2:** Patient related outcomes including KOOS subscale scores, IKDC (%) and EQ-5D VAS and Time-Trade-Off (TTO) score at first and final follow-up

Case	follow-up (years)	KOOS Symptoms %	KOOS Pain %	KOOS ADL%	KOOS Sport/Rec%	KOOS QoL %	KOOS total %	IKDC %	Equation 5D-VAS	Time-Trade-Off (TTO) score
1	4	79	64	75	25	13	51	54	45	
	8	79	81	87	70	31	69	63	80	0.36
2	4	82	69	79	35	19	57	51	65	
	8	82	92	99	0	25	59	62	90	0.99
3	2	43	58	97	20	81	60	53	95	
	7	68	97	99	55	38	71	68	80	0.89
4	1	96	100	100	85	69	90	79	85	
	6	100	100	100	80	81	92	100	100	1.00

### Case 1

A 12-year-old male (BMI 35.5 kg/m^2^) presented with a Schenck Type III lateral knee dislocation pattern following a sports-related fall. The injury complex comprised complete ruptures of the anterior cruciate ligament (ACL), posterior cruciate ligament (PCL), and the fibular-based lateral collateral ligament (LCL) complex, including avulsion of the biceps femoris tendon from the fibular head. Additionally, a traction injury of the common peroneal nerve was identified. (Fig. 1; Case 1) Surgical treatment was initiated single-stage: in the first procedure, arthroscopic primary repair of the ACL and PCL was performed, followed by open anatomical reconstruction of the LCL complex and reattachment of the biceps femoris tendon. During the initial operation, the peroneal nerve injury was assessed intraoperatively, but local conditions were not favorable for primary nerve reconstruction. In the postoperative course, insufficient neurological recovery became evident, prompting secondary microsurgical reconstruction using a sural nerve interposition graft. After 8 years the patient had an overall KOOS score of 69%, an IKDC of 63%. While the patient reported satisfactory knee function and pain levels in daily life and sporting activities, the patient reported a considerable psychological burden of the injury. The EQ-5D-5L-derived TTO utility score of 0.36 (Eq. EQ-5D VAS 80%) indicates a substantial reduction in health-related quality of life that was not proportionate to the patient’s favorable knee-specific functional scores.

### Case 2

A 14-year-old female (BMI 28.0 kg/m^2^) sustained a knee injury following a low-velocity torsional mechanism while standing on unstable sandy ground. Diagnostics revealed a Schenck Type III lateral knee dislocation pattern with ruptures of the ACL, PCL, and fibular-based LCL complex. Additionally, a dorsal capsular avulsion and an impression fracture of the medial femoral condyle were detected. (Fig. 1; Case 2) Definitive treatment was performed in a single-stage procedure, comprising ligament bracing of the ACL and PCL with simultaneous open anatomical reconstruction of the LCL complex. The observed delay between injury and first surgical intervention (12 days) was due to the initial admission of the patient to a peripheral hospital. At that institution, no definitive surgical treatment was performed, and the patient was subsequently referred and transferred to our center for further management. After 8 years the patient had an overall KOOS score of 59%, an IKDC of 62% and an EQ-5D VAS of 90%. While the patient did not report any significant pain (KOOS Pain 92%) or functional limitations in daily life (KOOS ADL 99%), the patient reported a KOOS Sport/Recreation subscore of 0%, indicating complete loss of participation in sporting and recreational activities, consistent with the significant limitations in sporting activity reported during the patient interview, mainly caused by the avoidance behavior. In addition to that the patient stated a considerable psychological burden caused by the injury, which may have contributed to this activity avoidance alongside any residual physical limitations. The EQ-5D-5 L-derived TTO utility of 0.99 (EQ-5D VAS 90%) indicates that the patient’s general health-related quality of life was preserved despite the pronounced sport-specific functional loss.

### Case 3

A 15-year-old male (BMI 26.5 kg/m^2^) sustained a high-energy knee injury in a road traffic accident (bike–tractor collision). The lesion was classified as a Schenck Type III lateral knee dislocation pattern, involving complete ruptures of the ACL, PCL and fibular-based lateral collateral ligament (LCL) complex, in combination with an impression fracture of the medial femoral condyle. (Fig. 1; Case 3) Surgical management was performed in a two-stage approach. Firstly, an external fixator was used for primary stabilization in an external hospital. In a second step after being transferred to our hospital arthroscopic ligament bracing of the PCL, transosseous suture repair of the ACL, and open anatomical reconstruction of the LCL complex was performed. After 7 years the patient had an overall KOOS score of 71%, an IKDC of 68%. While the patient only indicated minimal pain (KOOS pain of 97%) and limitations in daily life (KOOS ADL of 99%), the patient stated functional limitations during sporting and recreational activities. He was particularly limited in deep squatting and quick changes of direction. In addition to that the patient reported a significant psychological burden caused by the injury and reported a reduction of his quality of life (KOOS QoL 38%). The EQ-5D-5L-derived TTO utility of 0.89 (EQ-5D VAS 80%) indicates overall preserved general health-related quality of life despite this reduction in the disease-specific KOOS Quality of Life subscore.

### Case 4

A 16-year-old female (BMI 37.4 kg/m^2^) sustained a low-velocity knee injury while standing up from a cross-legged sitting position. The injury was classified as a Schenck Type III lateral knee dislocation pattern with complete rupture of the ACL, PCL and fibular-based LCL complex, accompanied by avulsion of the dorsal capsule, fibular avulsion of the biceps femoris tendon, impression fracture of the medial femoral condyle, and a medial meniscus root tear. (Fig. 1; Case 4) A two-stage surgical approach was undertaken. The first stage focused on restoration of the peripheral stabilizers and included arthroscopic medial meniscus root refixation, open anatomical reconstruction of the LCL complex, and reattachment of the biceps femoris tendon. The interval of approximately seven months between the two procedures was chosen to allow for soft tissue recovery, restoration of knee range of motion, and functional rehabilitation following peripheral stabilization. The second stage comprised reconstruction of the central stabilizers, including ACL and PCL reconstruction, which was performed once adequate knee function and local tissue conditions had been achieved. After 6 years the patient had an overall KOOS score of 92%, an IKDC of 100%. The EQ-5D-5L-derived TTO utility of 1.00 (EQ-5D VAS 100%) reflects a perfect health state, consistent with the excellent functional outcome despite the extended inter-stage interval in this case.

## Discussion

Tibiofemoral dislocations in the pediatric and adolescent population remain exceedingly rare, and evidence guiding their management is primarily extrapolated from adult literature or isolated case reports and case series [8, 11–13, 15]. This case series of four adolescent patients contributes to the limited body of data and highlights key considerations in the diagnosis, treatment, long-term patient-reported outcomes and health-related quality of life after these complex injuries.

In our case series, all the patients sustained Schenck Type III lateral dislocations, associated with both high- and low-energy trauma mechanisms. This pattern is consistent with existing literature on the epidemiology of tibiofemoral dislocations [7]. While this observation cannot be generalized given the small sample size, it raises the hypothesis that, in some pediatric and adolescent patients, severe multiligament injuries may occur even in the absence of high-energy mechanisms, and clinicians should therefore maintain a reasonably high index of suspicion irrespective of reported trauma intensity, particularly in patients with elevated BMI. Both patients in our series who sustained low-energy dislocations (Cases 2 and 4) had a BMI in the overweight-to-obese range (28.0 and 37.4 kg/m², respectively). This observation is consistent with prior evidence that obesity is associated with an increased likelihood of multiligamentous knee injury from low-energy mechanisms, potentially reflecting greater joint loading and reduced dynamic neuromuscular stabilization in patients with higher BMI [23, 24]. Early recognition is essential to initiate a timely and comprehensive diagnostic and therapeutic strategy.

Standardized early imaging, comprising MRI and vascular assessment, proved critical for comprehensive injury characterization and surgical planning in all patients. Consistent with previous reports, the observed injury patterns predominantly involved the ACL, PCL, and posterolateral corner structures, frequently accompanied by meniscal or chondral lesions, impression fractures, and capsular avulsions [8, 12, 15, 25, 26]. These findings are in line with pediatric multiligament knee injury cohorts, in which up to 55% of patients demonstrate associated meniscal or chondral damage [14, 15]. Neurovascular complications were identified in one of our cases, underscoring the importance of systematic peroneal nerve and vascular screening after pediatric knee dislocation—particularly following high-energy trauma or in the presence of perfusion abnormalities [8, 14, 25, 27, 28]. Given the substantial risk of missed injuries and long-term sequelae such as residual instability or growth disturbances, meticulous early evaluation remains essential in this population.

Surgical treatment in two out of four patients followed a staged approach, with initial stabilization followed by delayed ligamentous reconstruction. This strategy is in line with current adult trauma algorithms and appears both feasible and effective in the pediatric and adolescent population [1, 2, 8]. Importantly, no patient showed growth disturbances or angular deformities at the interim in-person clinical and radiological follow-up (see Methods); at final long-term follow-up (up to eight years), no patient reported subjective symptoms suggestive of angular deformity or leg-length discrepancy during the structured telephone interview, although this was not verified by repeat clinical or radiographic examination and must therefore be interpreted with caution (see Limitations).

The favorable functional outcomes observed in our series are in line with the limited existing literature on pediatric and adolescent multiligament knee injuries. (Table 3) Fanelli et al. reported good knee stability and a 55% return-to-preinjury-activity rate in 25 patients under 18 years after combined PCL, ACL, and collateral ligament reconstruction at a mean follow-up of 4.5 years [12]. Comparable results have been reported by Godin et al. and Lang et al. in adolescent cohorts, with most injuries caused by low-energy sports trauma and nearly all patients returning to their pre-injury activity level [13, 14]. A long-term case report by Oshima et al. further illustrated that even Schenck Type III injuries in skeletally immature patients can achieve complication-free return to competitive sports after single-stage primary ligament repair [11]. However, direct comparability with our series is limited: the Fanelli and Godin cohorts comprised predominantly patients above the age of 16, only a minority were skeletally immature, and none of these studies stratified outcomes by Schenck classification. (Table 3) To our knowledge, the present series therefore provides one of the longest follow-up periods (up to eight years) specifically for skeletally immature patients with only Schenck Type III dislocations. Importantly, the variability in subjective patient experience in our cohort, despite favorable KOOS and IKDC scores, highlights that functional recovery alone does not fully capture the impact of these injuries. Longitudinal analysis further illustrated that PROM trajectories do not necessarily move in parallel: in Case 3, KOOS symptom scores improved between the early and final follow-up, while KOOS Quality of Life subscores deteriorated substantially over the same period. Such divergence highlights the need to monitor multiple outcome dimensions over extended follow-up rather than relying on single composite scores.

**Table 3:** Overview of the current literature—tabular summary of studies reporting pediatric Schenck grade III or higher injuries. Only patients with Schenck grade III or higher injuries included in the studies are presented

Study	Year	*N*	Age (years)	Schenck Classification	Follow-up	Treatment	Key outcome
Oshima et al.	2017	1	14	KD III-M	8.5 years	Single-stage arthroscopic bicruciate ligament suture repair	unrestricted sports activities, knee ROM 0°-145°, Grade 1 posterior drawer, with inconstant and slight pain post-training, Lysholm score 95/100, Tegner score 9
Fanelli and Fanelli et al.	2016	25	16.7	KD III (M + L)	Mean 4.5 years	Combined PCL/ACL/collateral reconstruction	good knee stability in 65%; Lysholm score 93/100, 55% return-to-preinjury-activity rate
Godin et al.	2017	5	17.8 (2 aged 18, 2 aged 19 years)	KD III (M + L)	Mean 3 years	Combined PCL/ACL/collateral reconstruction	Nearly all patients returned to pre-injury activity level, Lysholm 86*, Tegner score 6*
Lang et al.	2023	5	14.4 (1 aged 18 years)	KD III-L	Mean 3.7 years	ACL and PCL reconstruction according to age, collateral ligament reconstruction or repair performed in cases of persistent varus or valgus instability.	Most patients returned to pre-injury activity level (data available for only 11 of 23 patients from the full cohort; the specific patients were not identified), Pedi-IKDC 81*, Lysholm 82.5*

* Data are presented for the entire study cohort; subgroup-specific data for patients with KD III injury or higher were not available

In three of our four patients, persistent psychological distress was reported despite favorable functional recovery. This discrepancy was most pronounced in Case 1, where moderate KOOS (69%) and IKDC (63%) scores contrasted with a TTO utility of 0.36, indicating a substantial reduction in health-related quality of life not captured by knee-specific PROMs. A related but distinct dissociation was observed in Case 2, who achieved an overall KOOS of 59% and a favorable TTO utility of 0.99, yet reported a KOOS Sport/Recreation subscore of 0%—indicating complete loss of participation in sporting and recreational activities. This finding illustrates that subscale-level analysis can reveal clinically important functional deficits that are masked by favorable composite or global HRQoL scores, and that the specific driver of such deficits (physical limitation, residual instability, or psychological avoidance) warrants targeted clinical inquiry at follow-up. Similar observations have been described in the broader pediatric orthopedic trauma literature, where post-traumatic stress, anxiety, and altered self-image are well-documented sequelae of major musculoskeletal injury [29, 30]. Taken together, these findings suggest that successful surgical and functional outcomes do not necessarily equate to complete patient recovery, and that routine psychological screening as well as access to psychosocial support should be integrated into follow-up protocols for pediatric patients after severe knee injuries.

This study has several limitations. First, the small sample size (*n* = 4) and the retrospective-prospective design limit the generalizability of our findings. Second, no pre-injury baseline data were available, and telephone-based follow-up introduces a potential for recall bias. Third, because all patients had reached legal adulthood at the time of outcome assessment, pediatric/adolescent-specific PROMs such as the Pedi-IKDC or KOOS-Child could not be applied at final follow-up, limiting comparability with other pediatric and adolescent series. Fourth, persistent psychological distress was identified using the EQ-5D-5L mental health dimension and unstructured patient narratives rather than validated psychometric instruments specifically designed to assess post-traumatic stress, anxiety, or depression (e.g., PHQ-9, KADS-11). This approach reflects routine clinical practice in our setting, where these commonly used PROMs are regularly collected during follow-up. However, these measures do not allow for a detailed or standardized assessment of psychological outcomes. Future studies are needed to determine to what extent such subdimensions reliably capture psychological burden compared to validated psychometric instruments and should incorporate these tools for more comprehensive evaluation. Lastly, no control group or comparable adult cohort from the same institution was analyzed. However, the rarity of the condition and the extended follow-up period of up to eight years provide valuable insights into a scarcely reported injury pattern. Future prospective, multicenter studies are needed to develop age-specific diagnostic and treatment algorithms for pediatric and adolescent tibiofemoral dislocations. Key unresolved questions include: whether single-stage versus staged reconstruction yields superior functional and psychosocial outcomes in skeletally immature patients; how inter-stage timing should be individualized to balance soft tissue healing against the risks of prolonged joint instability and stiffness; the comparative advantages and disadvantages of primary repair versus reconstruction, and of physeal-sparing versus transphyseal fixation techniques, with respect to growth preservation and mechanical stability; and which validated psychosocial screening instruments should be incorporated into routine follow-up to detect post-traumatic psychological distress in this population.

## Conclusion

This case series of four skeletally immature patients with tibiofemoral dislocations expands the limited pediatric and adolescent literature and provides one of the longest follow-up periods reported for Schenck Type III injuries. Severe multiligament injuries occurred after both high- and low-energy trauma, underlining the need for a high index of suspicion regardless of injury mechanism. Early standardized imaging and individualized surgical reconstruction strategies yielded favorable functional outcomes, with no growth disturbances observed at the last in-person clinical and radiological assessment and no subjective deformity reported at up to eight years. Notably, functional recovery did not uniformly translate into equivalent health-related quality of life: in some patients, favorable KOOS and IKDC scores coexisted with markedly reduced TTO utility values and persistent psychological distress. These findings suggest that the management of pediatric and adolescent tibiofemoral dislocations should extend beyond anatomical and functional restoration and should include routine psychosocial screening and support as an integral part of long-term follow-up.

## Data Availability

No datasets were generated or analysed during the current study.

## Abbreviations

TTO: Time Trade-Off
MRI: Magnetic resonance imaging
PCL: Posterior cruciate ligament
ACL: Anterior cruciate ligament
BMI: Body Mass Index
PROMs: Patient–reported outcome measures
IKDC: International Knee Documentation Committee score
KOOS: Knee injury and Osteoarthritis Outcome Score
EQ-5D-5L: EuroQol 5 dimension 5 level questionnaire
